# Factors associated with residents’ contract behavior with family doctors in community health service centers: A longitudinal survey from China

**DOI:** 10.1371/journal.pone.0208200

**Published:** 2018-11-29

**Authors:** Jiaoling Huang, Shanshan Liu, Rongrong He, Shuai Fang, Wei Lu, Jun Wu, Hong Liang, Yimin Zhang

**Affiliations:** 1 School of Social Development and Public Policy, Fudan University, Shanghai, China; 2 Pudong Institute for Health Development, Shanghai, China; 3 Shanghai Pudong Gongli Hospital, Shanghai, China; 4 School of Economics and Management, Hainan Normal University, Hainan, China; 5 Health and Family Planning Commission of Pudong New District, Shanghai, China; Sefako Makgatho Health Sciences University, SOUTH AFRICA

## Abstract

**Background:**

China adopted family doctor (FD) to help achieve “Healthy China 2030” through providing continuous, comprehensive, and life-cycle contract services. However, there is a disparity between actual and targeted FD use, as residents continue to visit specialists in large hospitals. The government implemented initiatives to improve residents’ willingness to sign up with and visit their FDs. Factors that influence contract behavior are therefore significant for frontier policy research.

**Methods:**

Two survey waves were conducted in Shanghai (2013 and 2016). The first wave included 2754 people and the second 1995 people. Exploratory factor analysis was used to synthesize “satisfaction” as a predictor of contract behavior. Pearson’s chi-square, pooled and logistic regression models were used to estimate associations between influencing factors and contract behavior, and clarify variations in factors across the two waves.

**Results:**

Four factors were extracted from 15 satisfaction items: “Treatment Environment,” “Medical Technology,” “Service Specification” and “Service Attitude”. Consistent with descriptive analysis, longitudinal analysis showed sociodemographic characteristics (age, education, marital status, and hukou) were significant predictors of contract behavior. The odds ratio of non-communicable diseases (NCD) patients for contract behavior was 2.218 times that of residents without NCD. Contract behavior was positively correlated with awareness of FD services (OR = 21.674, 95%CI = 15.043–31.229), satisfaction with Service Attitude (OR = 1.210, 95%CI = 1.009–1.451), and visit compliance (OR = 1.959, 95%CI = 1.564–2.452). Over time, the odds ratios of the married, Shanghai hukou, NCD, and awareness of FD services declined from 0.456, 1.795, 2.492, 28.690 to 0.443, 1.678, 1.910 and 14.031 respectively, while those of age, and visit compliance increased from 1.027, 1.521 to 1.041 and 2.305 respectively. In 2016, an education-contract gradient had formed (the higher the education level, the higher probability of signing with a FD), whereas high school education had the highest odds ratio (OR = 1.163,95%CI = 0.740–1.827) in 2013. Service Attitude was the only significant satisfaction-related predictor (OR = 1.358, 95%CI = 1.001–1.842) in 2016, compared with “Treatment Environment” (OR = 1.224, 95%CI = 1.001–1.496) and “Service Specification” in 2013(OR = 1.270, 95%CI = 1.040–1.552).

**Conclusions:**

Except for the socio-demographic variables, NCD, awareness of FD services, satisfaction and visit compliance were significant predictors of contract behavior with FDs. The effect of visit compliance had increased over time while NCD and awareness of FD services were losing impact over time. Significant satisfaction factors had also changed from “Treatment Environment” and “Service Specification” to “Service Attitude”.

## Introduction

The “Healthy China 2030” plan was released by the central government in Beijing in October 2016 and is a significant long-term national strategic plan [1]. The plan set out detailed arrangements for population health across a range of areas, including healthcare services, health environment, healthy lifestyles, and non-communicable disease prevention [2,3]. However, the plan’s core target and characteristics are distinguishable from previous plans in that it aims to improve population health by promoting healthy lifestyles, reducing disease incidence, and strengthening early diagnosis, early treatment, and early recovery. Such a massive health promotion campaign could be regarded as a preventive coping strategy for China’s rapidly aging society but also reflects the government’s concern about people’s health. The family doctor (FD) service, adopted as a fundamental healthcare policy following the medical reform in 2009 [4,5], is regarded as a significant tool to achieve Healthy China 2030.

Primary healthcare policies have been implemented in many countries (e.g., the United States, the United Kingdom, Canada, Australia, and Cuba) with diverse healthcare systems [6–10]. FD, also called the family physician or general practitioner, is intended to manage residents’ health, deal with common diseases, and curb healthcare expenses for both residents and governments [11–15]. The “gate-keeper” role of FD has been accepted by many governments, and confirmed by researchers globally [16–21]. For example, previous studies have indicated that primary healthcare supply is highly correlated with better health outcomes after basic demographic and socioeconomic variables are controlled, including total mortality, specific mortality, life expectancy, life span, and other health outcome indices (e.g., self-rated health) [22–27]. Other studies suggested that FD could save healthcare resources by managing medical cases as safely as other medical specialists, and generating positive net benefits for patients, the healthcare system, and society [28,29].

China adopted FD policy to provide basic healthcare to the whole population and help manage health and medical costs. In 2011, the State Council of the People’s Republic of China announced to establish FD system with Chinese characteristics. FD was designed to form the main body of tier-1 hospitals (the community health service center; CHSC). TheFD system was initially established in 2013 throughout the country. In 2015, the State Council released an important document that aimed to establish a referral system and strengthen the primary healthcare system [30]. However, most people continued their previous doctor visiting behavior, meaning they attended tier-2 and tier-3 hospitals and visited specialists regardless of their disease severity. Therefore, the government had to rethink its policy, and researchers had tried to explore and explain the disparity between policy targets and reality [31–33]. Lack of appeal for residents was identified as major problem. In 2016, the National Medical Reform Office released a new document that aimed to improve people’s willingness to visit FDs by contract services, and strengthen FDs’ enthusiasm to provide services through intrinsic motivation and external support [34]. This document set specific goals, including achieving a 30% contract rate by 2017 and covering the whole population by 2020.

To improve residents’ willingness to sign with FDs is the recent policy focus for the government, however, little research has concerned about this question [35], which is worth exploring for research and policy purposes. Recent studies in China mainly focused on the significance and the difficulties to establish FD system [36–39], or FD policy effect exploration including service utilization, non-communicable disease (NCD) management, medical expense, satisfaction, et al. [40–45]. Some researchers have paid attention to the significance of establishing a stable relationship with FDs or the contract status [46,47], however, contract behavior factor analysis was rarely explored. This study aimed to further explore factors associated with contract behavior to answer specific questions, as follows. 1) What are the demographic characteristics of contracted residents compared with non-contracted residents? 2) What are significant factors associated with contract behavior, after demographic variables are controlled? 3) Have these factors changed over time, and if so, how? Targeting at these research questions, we collected data from Shanghai of China, one of the earliest pilot cities practicing family doctor in China. The Shanghai Government announced to establish FD system in 2010. Seventy-four CHSCs from 10 regions were selected as FD reform pilots in 2011, and FD policy was implemented in all CHSCs in Shanghai from 2013.

## Materials and methods

### Ethical considerations

This study was approved by the academic ethics committee of Shanghai Pudong institute for health development. Our survey was voluntary, and residents could refuse to participate. All participants were asked to provide written consent before participating in this survey. All data were stored and processed anonymously. The entire research project was conducted by the Ethics Committee.

### Study sample and data collection

Data were collected with a questionnaire that targeted permanent residents aged 18 years and older living in one district in Shanghai, China. Permanent residents were defined as those had lived in the district for more than 6 months, regardless of registration type and location. Multistage cluster sampling was used to identify a sample of 3040 residents (10 sub-districts*four Neighborhood Committees*two communities*38 households*one resident/household), who were selected from the Residents Health Profile dataset at the Community Health Management Center. The “Health Profile” is a public health service that FDs must establish for every resident. After the sample was identified, 40 investigators (each investigator was responsible for one community) visited the selected residents, accompanied by Neighborhood Committee staff members that were familiar with the residents living in that community. All investigators were students majoring in sociology who had been trained before implementing the questionnaire survey. Three years later, new recruited investigators revisited the respondents from the first wave, also with the help of Neighborhood Committee staff.

A four-part structured questionnaire was designed covering demographic information, cognitive and contract behavior, CHSC service use, and satisfaction. Contract behavior was the key variable in our study. We marked a questionnaire as invalid if the contract behavior variable was missing. In the first wave (2013), 2754 valid questionnaires were collected. The second tracking survey was conducted 3 years later (2016) and tracked 1995 valid individuals (72.44%). Others were not followed up because of death, migration, being away on a long-trip, or being out-of-reach for other reasons.

### Variables

The key variable (contract behavior) was measured with the item “Did you sign with a family doctor?” (yes, no). Demographic variables included: age (years), gender (female, male), marital status (single, married, others), education level (primary school or lower; junior middle school; high school; bachelor’s degree, or higher), retired (yes, no), social insurance (yes, no), and hukou or household registration system (Shanghai, others). Data were also collected on: satisfaction, NCDs, visit compliance, and awareness of FD services. Satisfaction was assessed with fifteen items including medical equipment, medical environment, treatment approach, referral procedure et al., which could find in the attached questionnaire. All items were scored on a Likert scale from 1 (very dissatisfied) to 5 (very satisfied). The Cronbach’s alpha for this sample was 0.8744, indicating a good internal reliability. And subsequent Factor analysis results also revealed its structure validity by cumulative variation and other indexes. NCD was measured with the question “Do you suffer from any non-communicable diseases?” (yes, no). Visit compliance was measured with the item “Will you visit a family doctor when you are sick?” (yes, no), with responses labeled as high and low compliance, respectively. Finally, awareness of FD services was measured with the question “Do you know about family doctor contract services?” (yes, no).

### Statistical analysis

A descriptive analysis was conducted to describe the sample characteristics. Means and standard deviations were calculated for continuous variables, and frequencies for categorical variables. A comparison of sociodemographic characteristics between contracted and non-contracted residents was conducted using Pearson’s chi-square test. Exploratory factor analysis (EFA) was used to synthesize the satisfaction index. EFA was performed with principal component and varimax rotation. Sample adequacy was assessed with the Kaiser-Meyer-Olkin (KMO) method. After satisfaction indices were synthesized, they were included in models as independent variables. A pooled model was conducted for the longitudinal analysis, in which data from the two surveys were mixed and standard errors were adjusted for clustering [48]. Separate logistic regression models were performed for 2013 and 2016 to explore whether the effect of factors changed over time.

Survey data were entered into EpiData version 3.1 (The EpiData Association, Enghavevej 34, DK5230 Odense M, Denmark), and transferred to “.dta” format. All analyses were performed with STATA version 13.0 (StataCorp LP, Texas 77845 USA). A significance level of 0.05 was employed for all analyses.

## Results

### Sample characteristics

In total, 4749 residents were collected by the two survey waves. Evaluation of participants’ sociodemographic variables showed that most sociodemographic features did not change over time. Nearly 60% of respondents were female, 60% had a high school or above education, more than 70% were married, more than 90% had social insurance, and about 90% of residents were registered as Shanghai hukou (Table 1). Other characteristics did change; for example, the sample was ageing. The proportion of respondents aged over 60 years rose from 34% in 2013 to 62% in 2016, and the proportion of retirees rose from 54.83% to 70.98% (Table 1). In addition, the contract rate increased by 11% from 21.53% in 2013 to 32.53% in 2016 (Table 1). The 2017 policy goal set by the government was achieved in this sample.

**Table 1:** Distribution of respondents’ sociodemographic characteristics, 2013–2016.

Characteristics	2013 (N = 2754)	2016 (N = 1995)	All (N = 4749)
**Age, N(%)**			
18–45	757 (27.49%)	291 (14.59%)	1048 (22.07%)
45–60	776 (28.18%)	452 (22.66%)	1228 (25.86%)
60–70	820 (19.77%)	874 (43.81%)	1694 (35.67%)
70-	401 (14.56%)	378 (18.95%)	779 (16.41%)
Total	2754	1995	4749
**Gender, N(%)**			
Female	1647 (59.80%)	1254 (62.86%)	2901 (61.09%)
Male	1107 (40.20%)	741 (37.14%)	1848 (38.91%)
Total	2754	1995	4749
**Education Level, N(%)**			
Primary school or lower	293 (10.65%)	208 (10.52%)	501 (10.60%)
Junior Middle School	682 (24.79%)	562 (28.43%)	1244 (26.31%)
High School	914 (33.22%)	718 (36.32%)	1632 (34.52%)
Diploma degree or higher	862 (31.33%)	489 (24.73%)	1351 (28.57%)
Total	2751	1977	4728
**Marital Status, N(%)**			
Single	384 (13.97%)	147 (7.41%)	531 (11.22%)
Married	2029 (73.81%)	1576 (79.44%)	3605 (76.17%)
Others	336 (12.22%)	261 (13.16%)	597 (12.61%)
Total	2749	1984	4733
**Retired, N(%)**			
Yes	1510 (54.83%)	1416 (70.98%)	2926 (61.61%)
No	1244 (45.17%)	579 (29.02%)	1823 (38.39%)
Total	2754	1995	4749
**Social Insurance, N(%)**			
Yes	2556 (92.81%)	1887 (94.59%)	4443 (93.56%)
No	198 (7.19%)	108 (5.41%)	306 (6.44%)
Total	2754	1995	4749
**Hukou, N(%)**			
Shanghai	2409 (87.47%)	1823 (91.38%)	4232 (89.11%)
Others	345 (12.53%)	172 (8.62%)	517 (10.89%)
Total	2754	1995	4749
**Contract rate, N(%)**			
Yes	593 (21.53%)	649 (32.53%)	1242 (26.15%)
No	2161 (78.47%)	1346 (67.47%)	3507 (73.85%)
Total	2754	1995	4749

Note: Not all questions were answered by every respondent.

### Bivariate comparison between contracted and non-contracted residents

Bivariate comparisons of sociodemographic characteristics between contracted and non-contracted residents in both waves showed that contracted residents were generally older, less educated, had a higher proportion of retired people, and had Shanghai hukou (Table 2). This suggested age, education, retirement, and hukou were consistent in affecting contract behavior. The two groups of residents were also significantly different in marriage status. The contracted residents were in higher proportion in marriage rate in 2013, i.e. the marriage rate for the contracted and non-contracted were 77.66% and 72.75% respectively. However, the difference was reversed three years later, with the marriage rate 78.02% and 80.12% for the contracted and non-contracted groups (Table 2). Interestingly, social insurance lost its significance in the 3 years between the surveys. Contracted residents had a significantly higher proportion of social insurance in 2013, but social insurance characteristics could hardly be distinguished between the two groups in 2016 (Table 2).

**Table 2:** Distribution of contract rate stratified by sociodemographic characteristics, 2013–2016.

Sociodemographic Characteristics	2013(N = 2754)		2016(N = 1995)		All(N = 4749)	
	Non-contracted Residents	Contracted Residents	Non-contracted Residents	Contracted Residents	Non-contracted Residents	Contracted Residents
**Age**						
18–45	701(32.44%)	56(9.44%)	263(19.54%)	28(4.31%)	964(27.49%)	84(6.76%)
45–60	639(29.57%)	137(23.10%)	350(26.00%)	102(15.72%)	989(28.20%)	239(19.24%)
60–70	581(26.89%)	239(40.30%)	530(39.38%)	344(53.00%)	1111(31.68%)	583(46.94%)
70-	240(11.11%)	161(27.15%)	203(15.08%)	175(26.96%)	443(12.63%)	336(27.05%)
Total	2754		1995		4749	
Chi-2 (P value)	206.8166 (P = 0.000)		141.2306 (P = 0.000)		383.1417(P = 0.000)	
**Gender**						
Female	1284(59.42%)	363(61.21%)	828(61.52%)	426(65.64%)	2112(60.22%)	789(63.53%)
Male	877(40.58%)	230(38.79%)	518(38.48%)	223(34.36%)	1395(39.78%)	453(36.47%)
Total	2754		1995		4749	
Chi-2 (P value)	0.6252 (P = 0.429)		3.1895(0.074)		4.2124(P = 0.04)	
**Education Level**						
Primary school or lower	197(9.12%)	96(16.24%)	140(10.50%)	68(10.56%)	337(9.65%)	164(13.28%)
Junior Middle School	517(23.94%)	165(27.92%)	358(26.86%)	204(31.68%)	875(25.05%)	369(29.88%)
High School	716(33.15%)	198(33.53%)	483(36.23%)	235(36.49%)	1199(34.33%)	433(35.06%)
Bachelor degree or higher	730(33.80%)	132(22.34%)	352(26.41%)	137(21.27%)	1082(30.98%)	269(21.78%)
	2751		1977		4728	
Chi-2 (P value)	44.5487(P = 0.000)		8.1843(P = 0.042)		46.5787(P = 0.000)	
**Marital Status**						
Single	351(16.27%)	33(5.58%)	123(9.19%)	24(3.72%)	474(13.56%)	57(4.61%)
Married	1570(72.75%)	459(77.66%)	1072(80.12%)	504(78.02%)	2642(75.57%)	963(77.85%)
Others	237(10.98%)	99(16.75%)	143(10.69%)	118(18.27%)	380(10.87%)	217(17.54%)
Total	2749		1984			
Chi-2 (P value)	52.0419(P = 0.000)		36.9056(P = 0.000)		98.1200(P = 0.000)	
**Retired**						
No	1078(49.88%)	166(27.99%)	481(35.74%)	98(15.10%)	1559(44.45%)	264(21.26%)
Yes	1083(50.12%)	427(72.01%)	865(64.26%)	551(84.90%)	1948(55.55%)	978(78.74%)
Total	2754		1995		4733	
Chi-2 (P value)	90.0346(P = 0.000)		90.5138(P = 0.000)		208.6874(P = 0.000)	
**Social Insurance**						
No	176(8.14%)	22(3.71%)	77(5.72%)	31(4.78%)	253(7.21%)	53(4.27%)
Yes	1985(91.86%)	571(96.29%)	1269(94.28%)	618(95.22%)	3254(92.79%)	1189(95.73%)
Total	2754		1995		4733	
Chi-2 (P value)	13.7127(P = 0.000)		0.7622(P = 0.383)		13.2121(P = 0.000)	
**Hukou**						
Others	144(6.67%)	6(1.02%)	130(9.66%)	42(6.47%)	190(5.42%)	8(0.65%)
Shanghai	2016(93.33%)	585(98.98%)	1216(90.34%)	607(93.53%)	3134(94.58%)	1228(99.35%)
Total	2751		1995		4730	
Chi-2 (P value)	28.7487(P = 0.000)		5.6444 (P = 0.018)		52.0497(P = 0.000)	

### Multivariate analysis of contract behavior for pooled data

Table 3 presents the findings from principle component EFA with varimax rotation. Four factors were extracted with an eigenvalue >1.0 from the 15 satisfaction items, accounting for 75.44% of the variance (Table 3). The first factor explained 34.77% of the total variance and included f8, f9, f10, f11, f12, f13, f14, and f15, with each factor loading >0.45 (Table 3). The second factor explained 15.67% of the total variance and included f1, f2, and f5, with factor loadings >0.45 (Table 3). The third factor explained 12.83% of total variance and included f6 and f7, with each factor loading >0.45 (Table 3). Finally, the fourth factor explained 12.17% of the variance and included f3 and f4, with each factor loading >0.45(Table 3). No items cross-loaded on more than one factor. We labeled the four factors: “Treatment Environment,” “Medical Technology,” “Service Specification,” and “Service Attitude.” The dataset was suitable for factor analysis with KMO>0.5 (KMO = 0.888) and P<0.05 for Bartlett testChi2 = 10639.302 P = 0.000), indicating good construct validity for satisfaction scale. The Cronbach’s alpha for the satisfaction items was 0.8744, indicating the test had a good internal reliability (Table 3).

**Table 3:** Exploratory factor analysis results for satisfaction items.

Satisfaction Items	Factor1 (Treatment Environment)	Factor2 (Medical Technology)	Factor3 (Service Specification)	Factor4 (Service Attitude)	Uniqueness
f1	0.1984	**0.8668**	0.0809	0.0864	0.1954
f2	0.2621	**0.8160**	0.1487	0.128	0.2269
f3	0.1691	0.1683	0.1362	**0.8717**	0.1646
f4	0.128	0.0808	0.1191	**0.8992**	0.1543
f5	0.2561	**0.7537**	0.1499	0.1635	0.3171
f6	0.1154	0.0998	**0.9287**	0.1347	0.096
f7	0.1093	0.1217	**0.9321**	0.0919	0.096
f8	**0.7711**	0.2214	0.1345	0.2365	0.2824
f9	**0.7071**	0.2386	0.1870	0.2005	0.3679
f10	**0.7699**	0.1961	0.1385	0.1952	0.3115
f11	**0.8040**	0.1516	0.1375	0.1587	0.2865
f12	**0.8196**	0.1437	0.0748	0.0416	0.3004
f13	**0.8180**	0.1879	0.0552	0.068	0.2879
f14	**0.8272**	0.2139	0.0774	0.1111	0.2517
f15	**0.7832**	0.1910	0.0639	0.0357	0.3447
Variance	34.77%	15.67%	12.83%	12.17%	(75.44%)
Eigenvalue	5.23	2.35	1.93	1.83	--
KMO	0.888				
Bartlett test	Chi2 = 10639.302(P = 0.000)				

A pooled model was applied after satisfaction items were extracted. Table 4 showed the pooled estimators including both waves of data, indicating that age, education, marital status, and hukou were significant sociodemographic variables. However, “retired status” was not significant in the multivariate analysis. Specifically, the odds ratio of contract behavior increased on average by 3.4% for each year of increase in age (Table 4). Compared with those with primary school education or lower, the odds ratio for those with high school education and a bachelor’s degree or higher were 46% and 41% higher, respectively (Table 4). The odds ratio for married people and others were 45.3% and 54.1% of those who were single, respectively (Table 4). The odds ratio for residents with Shanghai hukou was 70.2% higher than for other registration (Table 4).

**Table 4:** Multivariate analysis of residents’ contract behavior, 2013–2016.

Variable	Odds Ratio	P Value	95% C.I.
**Age**	1.034	0.000	(1.023, 1.046)
**Gender**			
Female(Ref.)			
Male	0.997	0.974	(0.813, 1.222)
**Education Level**			
Primary school or lower(Ref.)			
Junior Middle School	1.269	0.165	(0.907, 1.776)
High School	1.460	0.030	(1.038, 2.054)
Bachelor degree or higher	1.410	0.066	(0.978, 2.035)
**Marital Status**			
Single(Ref.)			
Married	0.453	0.005	(0.261, 0.784)
Others	0.541	0.047	(0.295, 0.992)
**Retired**			
No(Ref.)			
Yes	0.930	0.609	(0.705, 1.228)
**Social Insurance**			
No(Ref.)			
Yes	1.388	0.128	(0.910, 2.118)
**Hukou**			
Others(Ref.)			
Shanghai	1.702	0.002	(1.213, 2.389)
**Suffer from NCD**			
No(Ref.)			
Yes	2.218	0.000	(1.813, 2.713)
**Do you know about FD service**			
No(Ref.)			
Yes	21.674	0.000	(15.043, 31.229)
**F1**	1.057	0.509	(0.897, 1.244)
**F2**	1.070	0.444	(0.900, 1.272)
**F3**	1.144	0.082	(0.983, 1.330)
**F4**	1.210	0.040	(1.009, 1.451)
**Visit compliance**			
Low(Ref.)			
High	1.959	0.000	(1.564, 2.452)
**Intercept**	0.002	0.000	(0.001, 0.004)
**Pseudo R**^**2**^	22.71%		
**Log likelihood**	-1396.0302		
**N**	2644		

NCD, awareness of FD, satisfaction with CHSC services, and visit compliance were also considered. These were all significant factors that influenced residents’ contract behavior. The odds ratio for signing with a FD among people with NCD was 2.218 times that of those without NCDs (Table 4). Those with high awareness of FD contract services were 21.674 times that of the low awareness ones to sign with a FD, and residents with higher satisfaction were also more likely to sign (Table 4). For each unit of increase in satisfaction with “Service Attitude,” the odds ratio increased by 21% on average (Table 4). Residents with high visit compliance were 95.9% more likely to sign with a FD than low compliance residents (Table 4).

### Separate multivariate analysis of contract behavior

Separate logistic models were applied for the two waves to investigate the third research question (changes in contract behavior over time). The results indicated that age, marital status, and hukou maintained a consistent effect on contract behavior. Specifically, the odds ratio for age had increased slightly, i.e. from 1.027 to 1.041 (Table 5); Compared with the single, the married were less likely to sign with FDs, and the signing probability for the single had increased slightly over three years (Table 5); Residents with Shanghai hukou were 1.795 times that of those from other provinces, and this odds ratio had decreased to 1.678 (Table 5); The impact of education had changed over the 3 years, with the highest odds ratio of signing with a FD in 2013 among those with high school education, followed by a bachelor’s degree and primary school or lower (with similar odds ratios). However, in 2016, an education-contract gradient had formed that showed residents with a higher education level were more likely to sign with a FD.

**Table 5:** Multivariate analysis of residents’ contract behavior in 2013 and 2016.

Variable	2013			2016		
	Odds Ratio	P Value	95% C.I.	Odds Ratio	P Value	95% C.I.
**Age**	1.027	0.000	(1.013, 1.041)	1.041	0.000	(1.026,1.056)
**Gender**						
Female(Ref.)						
Male	1.147	0.357	(0.857, 1.535)	0.876	0.357	(0.660,1.162)
**Education Level**						
Primary school or lower(Ref.)						
Junior Middle School	0.981	0.932	(0.626, 1.538)	1.618	0.056	(0.987,2.651)
High School	1.163	0.514	(0.740,1.827)	1.822	0.017	(1.114,2.981)
Bachelor degree or higher	1.087	0.738	(0.665,1.778)	1.826	0.026	(1.073,3.105)
**Marital Status**						
Single(Ref.)						
Married	0.456	0.033	(0.221,0.939)	0.443	0.041	(0.203,0.966)
Others	0.471	0.070	(0.208,1.064)	0.604	0.255	(0.253,1.439)
**Retired**						
No(Ref.)						
Yes	0.885	0.523	(0.609,1.286)	1.043	0.842	(0.689,1.580)
**Social Insurance**						
No(Ref.)						
Yes	1.774	0.107	(0.884,3.563)	1.247	0.490	(0.666,2.337)
**Hukou**						
Others(Ref.)						
Shanghai	1.795	0.020	(1.098,2.932)	1.678	0.030	(1.050,2.682)
**Suffer from NCDs**						
No(Ref.)						
Yes	2.492	0.000	(1.863,3.335)	1.910	0.000	(1.434,2.544)
**Do you know about FD service**						
No(Ref.)						
Yes	28.690	0.000	(17.184,47.901)	14.031	0.000	(8.118,24.253)
**F1**	1.224	0.049	(1.001,1.496)	0.828	0.190	(0.624,1.098)
**F2**	1.040	0.697	(0.854,1.267)	1.116	0.519	(0.799,1.558)
**F3**	1.270	0.019	(1.040,1.552)	1.023	0.848	(0.813,1.287)
**F4**	1.210	0.101	(0.963,1.520)	1.358	0.049	(1.001,1.842)
**Visit compliance**						
Low(Ref.)						
High	1.521	0.025	(1.055,2.193)	2.305	0.000	(1.728,3.075)
**Intercept**	0.002	0.000	(0.001,0.007)	0.001	0.000	(0.000,0.006)
**Pseudo R**^**2**^	27.60%			17.86%		
**Log likelihood**	-675.067			-709.305		
**N**	1396			1248		

All other related variables were significantly associated with contract behavior in both waves, but subtle changes were observed. The odds ratios for NCD and awareness of FD decreased from 2.492 and 28.690 to 1.910 and 14.031, respectively, while that for visit compliance increased from 1.521 to 2.305. The satisfaction factors “Treatment Environment” and “Service Specification” were significantly positively related to contract behavior in 2013, but “Service Attitude” was the only significant predictor in 2016 (Table 5).

## Discussion

Latest FD policy in China is focused on making FD services appealing to residents to improve residents’ willingness to sign with and visit FDs. Factors that may influence contract behavior are therefore significant for frontier policy research, although available field studies are inadequate.

Our research showed that the contract rate improved by 11% from 21.53% in 2013 to 32.53% in 2016 (Table 1). The contract rate target set by the government was 30% by the year 2017. Our findings were consistent with studies focused on the contract rate in China [49–50] that showed contract rates varied by region in China (20%–40%); however, most previous studies were conducted in Beijing, Shanghai, and Guangdong, where the FD policy was first piloted. The contract rate in pilot cities might be higher than other regions.

We conducted bivariate comparisons between contracted and non-contracted residents, and captured the sociodemographic characteristics of contracted residents. Specifically, contracted residents were older, had a lower education level (fewer people with a bachelor’s degree or above), had higher proportions of married people and those with social insurance, and most had Shanghai hukou. Results from previous studies were consistent with our findings. A study conducted in Guangdong Province indicated that patients with family practice contract care were more likely to be older, retired, and have NCDs [51]. Similar results were found in a previous Shanghai study conducted in 2011–2012, that showed contracted participants were older, had a higher probability of being retired, and were less healthy when compared with non-contracted participants [52]. Besides, several changes in socio-demographic characteristics were found across two waves. No significant difference in social insurance was found between the two groups in 2016. This could be explained by the fact that more people without social insurance were absorbed in the FD system. In addition, the marriage rate among contracted residents was significantly higher than among non-contracted residents in 2013, but this situation was reversed three years later. These characteristics provided clues as to influencing factors in the multivariable analysis.

Longitudinal analysis revealed accurate effects for the variables in the two survey waves. In accordance with the descriptive analysis, age, education, marital status, and hukou were significant sociodemographic variables. Specifically, the probability of signing with FD was positively correlated with age, education level, awareness of FD services, satisfaction with “Service Attitude,” and visit compliance, and was higher if residents were patients with NCDs or with Shanghai hukou. Similar previous studies were insufficient in exploring FD service contract behavior in China. Jing et al. analyzed the main factors influencing contract behavior in Pudong District, Shanghai [52]. That study drew on a sample of 1200 people who visited a FD in the six pilot CHSCs from 2011–2012, and used logistic regression including “year.” They found that age, education level, acceptance of CHSC, exposure to publicity, and year of investigation were significant. The term “publicity” was closely related to awareness of FD services. Another similar study was conducted in Shenzhen (China), involving 7761 CHSC patients [53]. The dependent variable used in that study was willingness to accept CHSC as gatekeeper. Multivariable analysis indicated that patients who were more familiar with the gatekeeper policy had higher level of willingness; conversely, reporting good health status was independently associated with decreased willingness. However, respondents in both studies were collected in CHSCs. The study sample could be biased as patients visiting CHSCs might be more familiar with, and more likely to recognize, sign with, and use FD contract services.

We applied subsequent separate logistic models to the two waves to explore variation in the effect of influencing factors. Age was maintained as a significant variable, and the education-contract gradient formed gradually (the higher the level of education, the higher the probability of signing with a FD). With the development of FD contracted services, more services were provided to residents including health examination, personalized health assessment and intervention, follow-up management for patients with NCDs, health education through a popular mobile phone application, home inpatient services, and convenient medicine delivery services [54–56]. This resident-centered service pack covered comprehensive health services rather than price discount, and might have been attractive to those in middle and upper socioeconomic status. We also observed that the coefficient of Shanghai hukou dropped from 1.795 to 1.678 over two waves (Table 5), indicating migrants were more willing to sign with FDs. The household registration system in China is highly correlated with social welfare [57,58]. Migrants, especially registered in rural areas, often live a marginalized life, are excluded from the urban social insurance system. They have less access to expensive urban healthcare systems, and have a disadvantaged health status [59–61]. The government announced that FD system was open to all permanent residents rather than the local residents. Access to the FD system may be the first step for those vulnerable people to utilize healthcare services equally with the Shanghainese.

Except for the sociodemographic characteristics, other related factors affecting variation were also observed. The odds ratio for patients with NCDs was 2.492 times that of their counterparts without such diseases to sign with FDs, and this coefficient declined to 1.910 in 2016 (Table 5). This was closely related to the policy implementation process. Older people, NCD patients, and poor people were initially targeted in the FD system [56], and preferential offers were available for patients with NCDs (i.e., FDs could prescribe 1–2 months’ medication for contracted patients with NCDs). As NCD patients were gradually covered by the FD system, the signing effect of this factor inevitably declined. The trend was similar for awareness of FD services. The FD contract service was new for residents at first, and awareness and acknowledge about FDs was low [62,63]. However, it still remained as a strong factor in 2016.

The effect of satisfaction and visit compliance on contract behavior had some interesting changes over time. “Treatment Environment” and “Service Specification” were significant factors influencing contract behavior in 2013. In 2016, “Service Attitude” was the only sensitive satisfaction factor. Establishing CHSCs facilities was a central government policy targeting to strengthen the primary healthcare system [64]. After infrastructure was established and irregular medical behavior eliminated, residents’ focus transferred to other aspects, such as service attitude. Our study suggested that residents paid more attention to whether they were treated in a friendly manner and well communicated with the FD in 2016 than in 2013. The only factor that continued to increase was visit compliance, whereas most other related variables lost impact. Visit compliance was a visit priority selection indicating people’s visiting preference when unwell. Those who visited CHSC as their priority choice were more likely to sign with a FD. Propaganda relating to FD services was suggested as an urgent measure in previous studies, and mass media tools, such as television, radio broadcasts, newspaper advertisements, blackboards, and lectures were proposed [52]. The government expected to improve awareness and promote ordered visiting behavior through visiting compliance. A FD-based referral healthcare system is expected to promote a well-constructed and rational functional healthcare system, lead more patients to CHSC for basic medical treatment, manage residents’ health through comprehensive healthcare services, reduce unnecessary medical resources waste, and improve people’s overall health.

## Conclusion

Residents’ contract rate for FD services increased by 11% from 2013 to 2016, indicating the preliminary target of a 30% contract rate was achieved. Sociodemographic characteristics including age, education, marital status, and hukou were significant predictors of contract behavior. NCD patients were in higher probability to sign with FDs. Contract behavior was positively correlated with awareness of FD services, satisfaction with “Service Attitude,” and visit compliance. Besides, the effect of visit compliance had increased over time while being NCD patients and awareness of FD services were losing impact over time. Significant satisfaction factors had changed from “Treatment Environment” and “Service Specification” to “Service Attitude”.

## Limitation

It is important to notice that several limitations still remained in our study. Longitudinal analysis performed better using at least three waves of data. Two waves of data had been collected in our study, and further survey will be conducted in the near future. Besides, we figured out the changes of influential factors over time, but factors might vary by regions. Data from different cities could be collected and hierarchical model could be applied.

## Supporting information

S1 File Questionnaire. (DOCX)
