# Supplementary material for: Factors associated with residents’ contract behavior with family doctors in community health service centers: A longitudinal survey from China
Source: PLoS One. 2018 Nov 29;13(11):e0208200. doi: 10.1371/journal.pone.0208200 (PMC6264849; doi:10.1371/journal.pone.0208200)
Supplement: S1 File — (DOCX) [file pone.0208200.s001.docx]

| **ID:** |
| --- |

**Family Doctor Contract Service Survey (2013/2016)**

(Permanent Residents)

**Introduction for investigators**: We conduct this survey to evaluate family doctor contract service, to further provide contract services which required by the residents and provide appropriate suggestions to the government.

**About Confidentiality:** You are free to accept or refuse this survey. Once you accepted, your answers will be kept safely and just used for academic use. The questionnaire is anonymous.

| **Is it the selected residents?**  **Yes, input the selected address:**  Sub-district__________, Neighborhood Committee__________, Building___________, Room___________.  **No, input the replaced address:**  Sub-district__________, Neighborhood Committee__________, Building___________, Room___________.  **Investigator ID:** ____________, **Signature:** _________________  **Visit date:** ________Year, _________Month, _________Date  **Quality of this questionnaire: Good Fair Bad**  **Check Operator ID:** _____________, **Signature:** ______________ |
| --- |

2018/7/8

**Part A. Demographic Information**

**A1** your birth date

_______ Year, _______Month, _______Date

**A2** your gender:

①female ②male

**A3** your nationality:

①Han ②Zhuang ③Manchu ④Hui ⑤Miao ⑥Uighur ⑦Yi ⑧Tujia ⑨Mongolian ⑩Korean ⑪Tibetan ⑫others

**A4** your education level:

①never received ②primary school graduated ③primary school graduation

④middle school graduation ⑤high school/secondary school/technical school

⑥college graduation ⑦undergraduate graduate ⑧master graduate, or higher

**A5** marital status:

①never married ②married ③cohabitation ④widowed ⑤divorced ⑥separated

**A6** Have you retired ?

①yes ②no

**A7** medical insurance you are participating in **(multiple choice):**

①Urban workers basic medical insurance ②Public medical care

③Urban residents medical insurance ④New rural cooperative medical care ⑤Commercial medical insurance ⑥others ⑦no participate ⑧not clear

**A8** hukou or household registration location:

①Shanghai ②others

**Part B. Cognitive and Contract Behavior**

**B1** Do you know or have heard of the family doctor?

①yes ②no

**B2** Do you know about family doctor contract services?

①yes ②no

**B3** Do you know the specific content of the family doctor service? **(multiple choice)**①not clear ②home treatment ③rehabilitation guidance ④family bed or care

⑤chronic disease or infectious disease visit ⑥elderly health care ⑦others

**B4** Did you sign with a family doctor?

①yes ②no

**B5** If you have not signed up yet, are you willing to accept the contract service?

①yes ②no ③not decided

**B6** Does any of your family member has signed with a family doctor?

①yes ②no

**Part C. Service Use**

**C1** Last time you were sick, what disease is it?

(Disease name)

**C2** Did you visit a medical institution for treatment this time?

①yes ②no

**C3** Which level of medical institution did you visit for this illness?

①Tertiary hospital ② Secondary hospital ③Community Health Center

**C4** If you visit the second or third level hospital, what is the reason? **(multiple choice)**

①feel the condition is serious ②advanced medical technology

③there are familiar specialist in the hospital ④others

**C5** If you visit the community health center, what is the reason? **(multiple choice)**

①feel the condition is lighter ②convenient for treatment ③cheaper

④good health care attitude ⑤others

**C6** Have you been referred to secondary and tertiary hospitals through the community health service center?

①yes ②no

**C7** Do you suffer from any chronic diseases?

①yes ②no

**C8** If you have a chronic disease, which of the following is it? How many years?

| Type | Suffering from the disease, check“√” | Illness years | Type | Suffering from the disease, check“√” | Illness years |
| --- | --- | --- | --- | --- | --- |
| hypertension |  |  | heart disease |  |  |
| diabetes |  |  | digestive disease |  |  |
| lung disease |  |  | tumor |  |  |
| stroke |  |  | arthritis |  |  |

**C9** What level of medical institutions do you usually visit for chronic diseases treatment?

①Community Health Center ②Secondary hospital ③Tertiary hospital

**C10** Will you visit a family doctor when you are sick?

①yes ②no ③depend on the situation

**Part D. Satisfaction**

**D1** Are you satisfied with the FDs’ ability to diagnosis accurately?

①very dissatisfied ②dissatisfied ③fair ④satisfied ⑤very satisfied

**D2** Are you satisfied with the FDs’ technical level?

①very dissatisfied ②dissatisfied ③fair ④satisfied ⑤very satisfied

**D3** Are you satisfied with the FDs’ patience when communicating with you?

①very dissatisfied ②dissatisfied ③fair ④satisfied ⑤very satisfied

**D4** Are you satisfied with the FDs’ carefulness in medical guidance and advice?

①very dissatisfied ②dissatisfied ③fair ④satisfied ⑤very satisfied

**D5** Are you satisfied with the validity of treatment approach?

①very dissatisfied ②dissatisfied ③fair ④satisfied ⑤very satisfied

**D6** Are you satisfied with the indiscriminate medicine prescribing phenomenon?

①very dissatisfied ②dissatisfied ③fair ④satisfied ⑤very satisfied

**D7** Are you satisfied with the indiscriminate medical examination phenomenon?

①very dissatisfied ②dissatisfied ③fair ④satisfied ⑤very satisfied

**D8** Are you satisfied with the convenience of CHSC services?

①very dissatisfied ②dissatisfied ③fair ④satisfied ⑤very satisfied

**D9** Are you satisfied with the humanized design of the treatment process

①very dissatisfied ②dissatisfied ③fair ④satisfied ⑤very satisfied

**D10** Are you satisfied with the medical equipment used?

①very dissatisfied ②dissatisfied ③fair ④satisfied ⑤very satisfied

**D11** Are you satisfied with the comfort level of the medical environment?

①very dissatisfied ②dissatisfied ③fair ④satisfied ⑤very satisfied

**D12** Are you satisfied with treatment privacy protection?

①very dissatisfied ②dissatisfied ③fair ④satisfied ⑤very satisfied

**D13** Are you satisfied with the referral procedure?

①very dissatisfied ②dissatisfied ③fair ④satisfied ⑤very satisfied

**D14** Are you satisfied with treatment duration (face-to-face with family doctor)?

①very dissatisfied ②dissatisfied ③fair ④satisfied ⑤very satisfied

**D15** Are you satisfied with the duration of waiting in line?

①very dissatisfied ②dissatisfied ③fair ④satisfied ⑤very satisfied

**Thank you for your participation !**
